# Radiological Impact of Atmospheric Nuclear Weapons Tests at Mururoa and Fangataufa Atolls to Populations in Oceania, South America and Africa: Comparison with French Polynesia

**DOI:** 10.31557/APJCP.2021.22.3.801

**Published:** 2021-03

**Authors:** Vladimir Drozdovitch, Florent de Vathaire, André Bouville

**Affiliations:** 1 *Division of Cancer Epidemiology and Genetics, National Cancer Institute, NIH, DHHS, Bethesda, MD, USA. *; 2 *National Institute for Health and Medical Research, Center for Research in Epidemiology and Population Health (CESP), INSERM U1018 / Gustave Roussy, Radiation Epidemiology Group, Villejuif, France. *; 3 *University Paris-Saclay, Villejuif, France.*; 4 *National Cancer Institute, Bethesda, MD, USA (retired). *

**Keywords:** Nuclear weapons, radiation, monitoring, Iodine-131, cancer

## Abstract

**Objective:**

To evaluate the potential radiological impact of atmospheric nuclear weapons tests conducted in 1966-1974 at Mururoa and Fangataufa atolls on populations in Oceania, South America and Africa.

**Methods:**

Results of measurements of total beta(β)-concentrations in filtered air and ^131^I activity concentrations in locally produced cow’s milk in Oceania, South America and Africa after the tests were compared with those in French Polynesia. Radiation doses due to external irradiation and thyroid doses due to ^131^I intake with milk by local populations were also compared.

**Results:**

Higher total β-concentrations in filtered air, ^131^I activity concentrations in locally produced milk and radiation doses to local population were, in general, observed in French Polynesia than in other countries in the southern hemisphere. However, for specific years during the testing period, the radiological impact to South America was found to be similar or slightly higher than that to Tahiti. The resulting thyroid doses in the considered countries were lower than those in French Polynesia with two exceptions: thyroid doses due to ^131^I intake with cow’s milk for 1-y old child in 1968 were higher in Peru (0.35 mGy) and in Madagascar (0.30 mGy) than in Tahiti (0.25 mGy). However, the populations outside French Polynesia received doses lower than those from the natural sources of radiation.

**Conclusion:**

According to the current knowledge in radiation epidemiology, it is very unlikely that nuclear fallout due to French nuclear tests had a measurable radiological and health impact outside French Polynesia.

## Introduction

In July 1962, France decided to move its nuclear tests from Reggane and Taourirt Tan Afella in the south of Algeria to the atolls of Mururoa and Fangataufa of the Tuamotu-Gambier archipelago in French Polynesia. The first atmospheric nuclear weapons test took place four years later, on 2 July 1966, i.e. 3 years after the Partial Test Ban Treaty was signed on 5 August 1963 by the Soviet Union, the United Kingdom, and the United States. A total of 41 atmospheric tests, excluding 5 safety tests, and of 147 underground tests were performed in these atolls, the last one taking place in 27 January 1996.

Because local radioactive fallout of atmospheric nuclear tests can, in some situations, contaminate areas up to several thousands of kilometres from the test site (Bouville, 2020), this decision faced a strong opposition from the populations of neighbouring countries. This opposition continued during the entire period of the tests, included calls for boycott of French products (Holdstock, 1995), culminated with the sabotage of the Greenpeace Rainbow Warrior ship by the French Army on 10 July 1985, and with the announcement, of the resumption of the tests by Jacques Chirac on 13 June 1995 (Danielson, 1993; Willis, 2006). 

Australia, New Zealand and some countries in South America (Argentina, Brazil) set up radiation monitoring laboratories in order to collect information on radioactive fallout following the tests and to document their potential impact on neighboring populations. During the atmospheric nuclear weapons tests period, these laboratories, as well as the French ones based in French Polynesia and in other French Overseas territories (French Guiana, Madagascar, New Caledonia, Réunion, Wallis and Futuna) as well as in South America (Bolivia, Chile, Colombia, Ecuador, Peru), collected information on radioactive contamination of the environment that was regularly sent to the United Nations Scientific Committee on the Effects of Atomic Radiation (RF, 1967-1975).

In the framework of a case-control study on differentiated thyroid cancer risk factors in French Polynesia, we have documented the nuclear fallout, the radiation dose to the thyroid gland (Drozdovitch et al., 2008), and the resulting effects on thyroid cancer on French Polynesia (de Vathaire et al., 2010). This first study was based on French scientific reports to UNSCEAR, which provided, for several localities in each of the archipelagos of French Polynesia, total beta(β)-activities in filtered air, total gamma(γ)-activities in local foodstuffs, as well as ^131^I and ^137^Cs concentrations in fresh cow’s milk produced in Tahiti, and a few results of measurements of exposure rates and deposition densities in Gambier, Tuamotu atolls and Tahiti. Nevertheless, for reasons of national security during this period, these reports included only summarized data that impacted the credibility of the results (Tubiana and Aurengo, 2007; Pitrou, 2015).

Thanks to the decision of French Government to declassify in 2013 all original reports of the radiation protection services in charge of radiation monitoring during the atmospheric nuclear tests, we were able, in the framework of an extension of the case-control study on thyroid cancer risk factors, to perform a more documented estimation of the magnitude of the radioactive fallout throughout French Polynesia and of the thyroid radiation doses received by French Polynesians (Drozdovitch et al., 2020; Drozdovitch et al., 2021). 

Nevertheless, up to now, no publication has documented the health impact of French nuclear tests on the populations of neighboring countries, in particular of Oceania (Cook Islands, Fiji, New Caledonia, Samoa, Tonga, Wallis and Futuna), New Zealand and Australia located at distances from 2,150 to 6,900 km to the west of the nuclear test sites in French Polynesia, and of South America (Bolivia, Chile, Colombia, Ecuador, French Guiana, Peru) located at distances from 6,600 to 9,700 km to the east of the nuclear test sites. We took therefore the opportunity of these investigations to evaluate the radiation exposures of these populations in order to better understand their potential health impact. 

## Materials and Methods

*Radiation monitoring*

Radiation monitoring in Oceania, South America and Africa during the 1966‒1974 time period of atmospheric nuclear weapons tests in French Polynesia was conducted in: 

– New Zealand, Cook Islands, Fiji, Samoa, and Tonga by the National Radiation Laboratory (NRL, New Zealand);

– Australia by the Atomic Weapons Tests Safety Committee (AWTSC, Australia);

– Bolivia, Chile, Colombia, Ecuador and Peru by national organizations of these countries in cooperation with French authorities (Coulon et al., 2009);

– French overseas territories, French Guiana, New Caledonia, Réunion Island, Wallis and Futuna, and Madagascar. 

**Figure 1:**
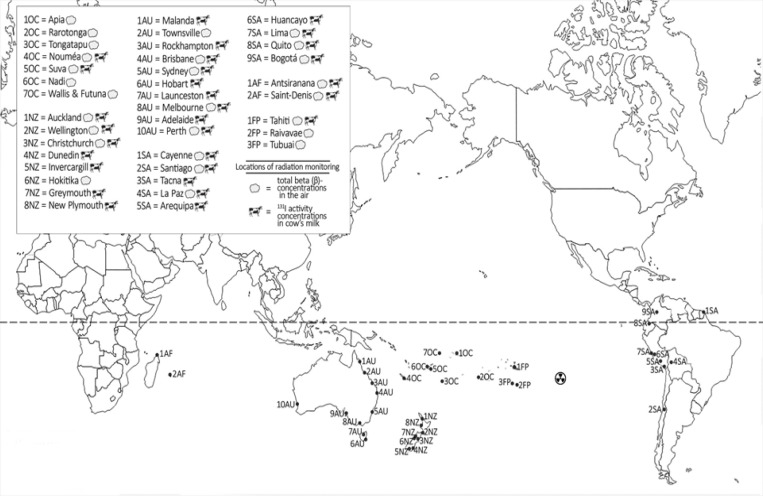
Geographical Pattern of Network for Fallout Monitoring in Oceania Islands (OC), New Zealand (NZ), Australia (AU), South America (SA) and Africa (AF) during the Period of Atmospheric Nuclear Tests in French Polynesia (FP).

Radiation monitoring included, among others, measurements of total β-concentrations in filtered air and ^131^I activity concentrations in locally produced cow’s milk, which are the most relevant to the estimation of thyroid doses to populations, as well as precipitation, daily or weekly, at some monitoring stations. Figure 1 shows the geographical distribution of the fallout monitoring network in Oceania, South America and Africa during the time period of the atmospheric nuclear tests in French Polynesia. Monitoring stations in Oceania and Africa were located west / south-west of the nuclear test sites on Mururoa and Fangataufa atolls in French Polynesia at distances ranging from 2,150 km (Rarotonga, Cook Islands) to 16,100 km (Antsiranana, Madagascar), while the monitoring stations in South America were located east of the test site at distances from 6,600 km (Lima, Peru) to 9,700 km (Cayenne, French Guiana). Except for Cayenne and Bogotá, located at a latitude of about 5^o ^latitude North, all monitoring stations are situated in the southern hemisphere, although Quito is very close to the equator (0.18^o^ latitude South).

Radiation monitoring in French Polynesia and in other French overseas territories during the 1966‒1974 was conducted by two organizations: the Joint Radiological Safety Service (Service Mixte de Sécurité Radiologique, SMSR), which was in charge of the radiation measurements in the physical environment (exposure rates, concentrations in air and water, and deposition on the ground), and the Joint Biological Control Service (Service Mixte de Contrôle Biologique, SMCB), which was in charge of the radiation measurements performed in the biological environment (plants, vegetables, fruit, milk, milk products, animals from the terrestrial and aquatic environments) (Coulon et al., 2009; MDF, 2009). A more detailed description of the radiation monitoring network in French Polynesia can be found elsewhere (Drozdovitch et al., 2020).

Table 1 compares the numbers of measurements that were done in Oceania (NRL, 1966-1974; Bonnyman and Duggleby, 1967-1969; Gibbs et al., 1967-1969; RF, 1967-1975; AWTSC, 1971-1974), South America and Africa (RF, 1967-1975) with those done in French Polynesia (Drozdovitch et al., 2020) during the monitoring period. The number of measurements of total β-concentration in air that was done in French Polynesia is similar to that in Oceania, 7,526 vs. 9,018, respectively and much higher than that in South America (933 measurements) and in Africa (279 measurements). The number of measurements of ^131^I activity in cow’s milk is much lower for French Polynesia than for Oceania, 482 vs. 12,449, and for South America (1,118 measurements), because cow’s milk in French Polynesia was only produced in Tahiti. 

*Time of arrival of fallout*

**Figure 2:**
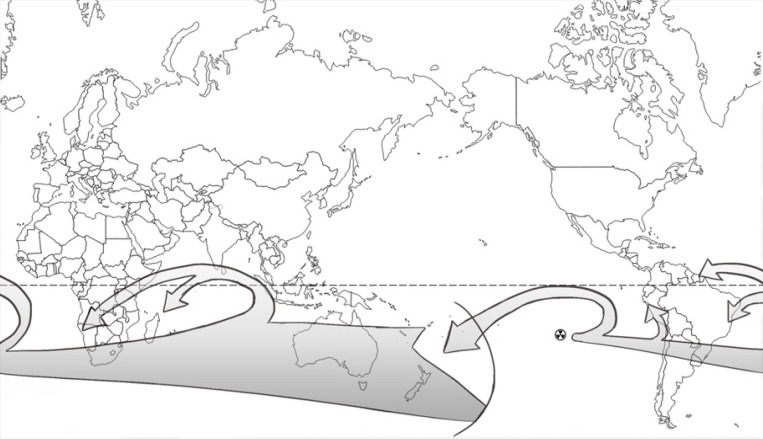
Typical Trajectories of ‘Primary’ and ‘Secondary’ Clouds in the Southern Hemisphere that Moved to Areas from the Nuclear Test Sites Following Atmospheric Nuclear Weapons Test at Mururoa and Fangataufa Atolls

The radioactive clouds produced by the nuclear explosions usually extended vertically to the highest levels of the troposphere. They were then transported by the local winds, which at Mururoa and Fangataufa generally blew toward the East and were affected by high-pressure anticyclonic zones located to the North and to the South. The bulk of the radioactive clouds, named here ‘primary’, consequently blew between 10º and 40º of latitude South in the general direction: South America → Africa → Australia → South Pacific. However, parts of the radioactive clouds, under the influence of the high-pressure systems, were extracted from the ‘primary’ clouds, changed direction, and in some cases, led to ‘secondary’ clouds that moved to areas west of the nuclear test sites, where most of the atolls and islands of French Polynesia and Oceania are located. Figure 2 shows typical trajectories of ‘primary’ and ‘secondary’ clouds in the southern hemisphere following atmospheric nuclear weapons tests at Mururoa and Fangataufa atolls.

Taking the test Aldebaran as a typical example, the ‘primary’ cloud reached the western coast of South America 5 days after the test, the western coast of Africa 10 days after the test, Australia 14 days after the test, and New Zealand 16 days after the test; the ‘primary’ cloud completed its first pass around the world in about 20 days (RF, 1967); in addition, three ‘secondary’ clouds were generated: (1) the first one, formed before the ‘primary’ cloud reached South America, led to the contamination of the South Pacific Islands 11 to 13 days after the test, (2) the second one, generated before the ‘primary’ cloud reached Africa, resulted in fallout over the north-eastern part of Brazil 12 days after the test and in French Guiana 17 days after the test, and (3) a third one, formed while the ‘primary’ cloud was in the proximity of Australia, led to the contamination of Réunion Island and Madagascar 17 days after the test. It is remarkable that the ‘primary’ cloud reached Australia and New Zealand, located about 28,000 km away from the test site along the route taken by the ’primary’ cloud, just a few days before the first ‘secondary’ cloud arrived in the South Pacific Islands, located at much closer distances of about 4,000 km from the test site; along the same lines, the contamination of the monitoring stations located in Africa (Réunion Island and Madagascar) occurred a few days after the ‘primary’ cloud reached Australia and New Zealand, although these countries are about 22,000 km apart along the route taken by the ’primary’ cloud. 

For most monitoring stations, the mode of the distribution of the times of arrival (TOA) of fallout was in the 10–to–19.9 days bin, with an average frequency of 50% and a range from 35 to 75%. There were five exceptions: Chile and Bolivia, where the modes were in the 5–to–9.9 days bin, and Australia, New Zealand, and French Guiana, where the modes of the distributions were greater than 20 days. For Tahiti (French Polynesia) and Santiago (Chile) the percentage of tests with TOA≤5 d was greater than 20%, thus resulting in smaller average TOAs for these locations than for any other monitoring station considered in this paper.

*Dose estimates*

Radiation doses due to external irradiation and intake of ^131^I with cow’s milk were estimated for the population of Australia by AWTSC (1971-1974) and Gibbs et al., (1967-1969). Thyroid doses due to ^131^I intake with cow’s milk to 1-y old child in South America and Africa were estimated by RF (1967-1975). Thyroid doses to 1-y old children in New Zealand were extracted from UNSCEAR (1977).

For Fiji (Suva), Samoa (Apia), Tonga (Tongatapu) and Cook Islands (Rarotonga) thyroid doses were estimated in this study for adults and 1-year old children for the following pathways of exposure:

– Inhalation of ^131^I and of short-lived radioiodine isotopes (^132^I, ^133^I and ^135^I) and radiotellurium (^132^Te) with contaminated air;

– Ingestion of ^131^I with fresh cow’s milk;

– External irradiation from radionuclides deposited on the ground and other materials.

A detailed description of the methods used to calculate thyroid doses for the populations of these islands can be found elsewhere (Drozdovitch et al., 2021). Behaviour and age-specific consumption rates of cow’s milk by residents of Fiji and Samoa were assumed to be the same as those for population of Tahiti (Drozdovitch et al., 2019).

## Results

*Time-integrated total β-concentration in air*

Table 2 compares the time-integrated total β-concentrations in air measured in Oceania, South America, and Africa (NRL, 1966-1974; RF, 1967-1975) following atmospheric nuclear weapons tests conducted at Mururoa and Fangataufa atolls in 1964-1974 with those measured in Tahiti (French Polynesia) (Drozdovitch et al., 2020). The lowest β-concentrations in air were observed in the southern hemisphere in 1972 and 1973 following nuclear test series with low fission yield. The highest total β-concentrations in air were observed, in general, in Tahiti for each year of testing. Time-integrated total β-concentrations in air in Fiji, Samoa, Tonga and Cook Islands were found, in most years, to be less than those in Tahiti by factors from 50 to 200. This factor varied, for most years and monitoring locations, from around 100 to 1,100 for New Zealand, from 10 to 800 for South America, and from 5 to 550 for Africa. The smallest values of the factor were found for the testing campaigns of 1968, 1970, 1971 and 1973.

These results could be expected, because of radioactive decay of radionuclides, as well as dilution and depletion of radioactive clouds during their transfer over distances of 1,000 km (between Tahiti and Rarotonga), 3,400 km (between Tahiti and Fiji), 4,300 km (between Tahiti and Wellington, New Zealand), and remarkable distance, from 6,600 km to 16,100 km, between test site and monitoring locations in South America and Africa. 

^131^ *I activity concentration in locally produced cow’s milk*

Table 3 compares the time-integrated ^131^I activity concentrations in cow’s milk locally produced in 1966-1974 in Oceania (NRL, 1966-1974; Bonnyman and Duggleby, 1967-1969; Gibbs et al., 1967-1969; AWTSC, 1971-1974), South America, Madagascar, and French Polynesia (RF, 1967-1975). Results for Réunion Island (Saint-Denis) are not shown as ^131^I activity concentrations in milk were reported for all measurements to be less than the limit of detection, which was typically 0.6-0.7 Bq L^-1^. 

The time-integrated ^131^I activity concentration in cow’s milk produced in Australia (Malanda), Peru (Lima, Tacna and Arequipa), Chile (Santiago), Bolivia (La Paz) and Madagascar (Antsiranana) in some years during 1966-1972 was similar or even higher than that produced in Tahiti. In 1973-1974, the time-integrated ^131^I activity concentration in cow’s milk produced in Tahiti was found to be higher than the concentrations in other monitoring stations in the southern hemisphere by factors from 2.3 to around 1900.

*Radiation doses to local populations*

Table 4 compares annual doses due to external irradiation to population of Australia (AWTSC, 1971-1974) and thyroid doses due to ^131^I intake with cow’s milk for 1-y old child from Australia (Gibbs et al., 1967-1969; AWTSC, 1971-1974), New Zealand (UNSCEAR, 1977), South America and Madagascar (RF, 1967-1975) with those from Tahiti (Drozdovitch et al., 2021). Thyroid doses due to ^131^I intake with cow’s milk, which were taken from AWTSC and RF reports, were calculated for daily consumption of cow’s milk by 1-y old child of 0.7 L for Australia, South America and Madagascar; daily consumption of cow’s milk for Tahiti was 0.37 L (Drozdovitch et al., 2019).

Doses due to external irradiation to population of Tahiti were found to be higher than those in Australia by factor from 1.7 in 1971 to 64 in 1974 (Table 4). It should be noted that doses due to external irradiation for population of Australia were estimated without account for shielding properties of buildings and environment which would reduce exposure by factor from 2 (rural areas) to 3 (urban areas).Thyroid doses due to ^131^I intake with cow’s milk for 1-y old child were also found to be the highest in Tahiti, except in 1968 when dose for Tahiti residents (0.25 mGy) was lower than that for Antsiranana, Madagascar (0.30 mGy) and for Peru (0.35 mGy). However, in general these thyroid doses were very low. 

Table 5 compares the thyroid doses from different exposure pathways calculated in this study for a representative 1-y old child and a representative adult who resided in Fiji, Samoa, Tonga, Cook Islands and Tahiti after test Centaure on 17 July 1974. Total thyroid doses due to all pathways to population of Tahiti was higher than those to population of islands in Oceania by factor varying from 20 to around 750. The highest thyroid doses in Oceania were estimated for population of Samoa; however, these doses were less than those in Tahiti by factor of 20 and 35 for to 1-y old children and for adults, respectively. 

Thyroid doses received outside French Polynesia by children born during the French atmospheric nuclear test period were lower than 1 mGy. Annual effective doses from the natural sources of radiation (cosmic rays and terrestrial radiation) were estimated to be around 1.7 mSv to populations of Oceania and even higher to populations of South America (UNSCEAR, 2010). Therefore, the potential health impact of doses lower than 1 mGy from the French atmospheric nuclear tests has to be related to results of the largest pool of cohorts that aimed to evaluate the effects of low radiation doses (<100 mGy) to thyroid during exposure in childhood (Lubin et al., 2017). In this pool, a mean dose of 2 mGy has been associated to an increase of 0.4 case in a group of 9,464 subjects cumulating 367,606 person years of follow-up, corresponding to an excess of 1 case of thyroid cancer in 100,000 persons in 10 years.

**Table 1:** Number of Measurements Done in Oceania, South America, Africa and French Polynesia in 1966-1974

	Country	Number of measurements done in								Total
		1966	1967	1968	1970	1971	1972	1973	1974	
Total β-concentration in air	Fiji	184	242	280	327	275	202	212	267	1,989
	Samoa	–	121	148	167	148	103	112	136	935
	Wallis and Futuna	–	–	–	–	16	12	12	14	54
	Tonga	–	–	145	165	149	101	112	136	808
	Cook Islands	181	–	–	–	–	–	–	134	315
	New Zealand ^a^	538	362	442	667	594	367	403	546	3,919
	New Caledonia	21	21	21	24	15	12	12	7	133
	Australia ^b, c^	790	12	112	–	–	–	–	–	914
	South America	89	85	76	99	82	59	60	83	633
	Africa	40	41	42	48	30	24	24	30	279
	French Polynesia ^d^	1,096	220	1,175	1,188	992	856	938	1,061	7,526
										
^131^I activity in cow’s milk	Fiji	5	9	33	49	49	36	42	60	283
	Samoa	6	18	30	45	41	25	36	45	246
	New Zealand ^e^	378	137	303	366	377	249	231	380	2,421
	New Caledonia	–	15	35	3	–	11	21	28	113
	Australia ^c, f^	1,392	648	454	592	536	288	244	396	4,550
	South America	31	110	115	264	132	204	91	171	1,118
	Africa	4	20	45	8	5	25	32	5	144
	French Polynesia ^g^	–	64	68	74	89	13	44	130	482

^a^, Auckland, Wellington and Christchurch in 1966-1968. Hokitika was added in 1970; ^b^, Melbourne, Sydney, Brisbane, Townsville, Perth in 1966 and 1968; Melbourne in 1967; ^c^, Number of measurements was estimated based on reported number of collection stations, duration of monitoring period and frequency of sample collection; ^d^, Multiple locations, see Drozdovitch et al. (2020) for details; ^e^, Auckland, New Plymouth, Wellington, Greymouth, Christchurch, Dunedin and Invercargill; ^f^, Perth, Adelaide, Melbourne, Hobart-Launceston, Sydney, Brisbane, Rockhampton and Malanda; ^g^, Tahiti.

**Table 2:** Time-Integrated β-Concentration in Air Measured in Oceania, South America, Africa and French Polynesia in 1964-1974

Year		Testing period ^a^		Monitoring period ^a^	Time-integrated β-concentration in air (Bq s m^-3^)																	
					Fiji (Nadi)		Fiji (Suva)		Samoa (Apia)		Wallis and Futuna		Tonga (Tongatapu)		Cook Islands (Rarotonga)		New Zealand ^b^		New Caledonia (Nouméa)		French Polynesia (Tahiti)	
1966		03/07-05/10		01/07-31/12	8.1×10^5^		–		–		–		–		3.4×10^5^		1.2×10^5^		2.7×10^5^		9.6×10^6^	
1967		06/06-03/07		01/06-30/09	1.5×10^5^		1.5×10^5^		4.4×10^5^		–		–		–		3.4×10^4^		8.6×10^4^		7.1×10^6^	
1968		08/07-09/09		04/07-30/11	4.7×10^5^		5.7×10^5^		6.4×10^5^		–		3.8×10^5^		–		8.8×10^4^		3.0× 10^5^		1.4×10^6^	
1970		16/05-07/08		16/05-31/10	2.8×10^5^		3.3×10^5^		5.7×10^5^		–		3.2×10^5^		–		9.8×10^4^		1.8×10^5^		1.1×10^6^	
1971		05/06-14/08		04/06-31/10	2.7×10^5^		3.5×10^5^		9.1×10^5^		1.9×10^5^		3.6×10^5^		–		1.3×10^5^		1.6×10^5^		1.8×10^6^	
1972		25/06-27/07		20/06-30/09	1.3×10^4^		1.8×10^4^		1.1×10^4^		3.1×10^3^		1.6×10^4^		–		1.0×10^4^		9.4×10^3^		1.6×10^5^	
1973		21/07-28/08		12/07-31/10	1.4×10^4^		1.8×10^4^		9.2×10^5^		3.3×10^5^		3.2×10^4^		–		5.7×10^3^		2.0×10^3^		1.2×10^6^	
1974		16/06-14/10		17/06-31/10	2.9×10^5^		3.1×10^5^		2.8×10^6^		1.6×10^6^		3.3×10^5^		1.2×10^6^		5.0×10^4^		1.5×10^5^		5.5×10^7^	
Total					2.3×10^6^		1.7×10^6^		6.3×10^6^		2.1×10^6^		1.4×10^6^		1.5×10^6^		5.4×10^5^		1.2×10^6^		7.7×10^7^	
Year	Testing period ^a^		Monitoring period ^a^			Time-integrated β-concentration in air (Bq s m^-3^)															
						Ecuador (Quito)		Peru (Lima)		Colombia (Bogotá)		Chile (Santiago)		Bolivia (La Paz)		French Guiana (Cayenne)		Madagascar (Antsiranana)		Réunion Island (Saint-Denis)	
1966	03/07-05/10		01/06-31/12			9.2×10^4^		1.8×10^6^		7.0×10^4^		1.8×10^6^		–		2.7×10^5^		5.5×10^5^		3.0×10^5^	
1967	06/06-03/07		01/06-31/12			4.0×10^4^		3.6×10^5^		1.9×10^4^		2.4×10^5^		–		6.7×10^4^		1.3×10^5^		1.1×10^5^	
1968	08/07-09/09		01/06-31/12			1.8×10^5^		7.2×10^5^		1.6×10^5^		6.2×10^5^		–		2.7×10^5^		3.4×10^5^		2.9×10^5^	
1970	16/05-07/08		01/05-31/12			7.2×10^4^		1.9×10^6^		5.8×10^4^		6.9×10^5^		9.3×10^5^		1.0×10^5^		1.9×10^5^		2.4×10^5^	
1971	05/06-14/08		01/06-31/10			5.3×10^4^		9.0×10^5^		1.3×10^5^		6.7×10^5^		3.5×10^5^		5.8×10^4^		1.4×10^5^		2.2×10^5^	
1972	25/06-27/07		01/06-30/09			8.3×10^3^		1.3×10^5^		7.1×10^4^		3.7×105		3.9×10^4^		3.6×10^3^		4.8×10^3^		1.2×10^4^	
1973	21/07-28/08		01/07-31/10			4.7×10^3^		–		1.2×10^4^		3.2×10^5^		2.4×10^5^		9.3×10^3^		2.9×10^3^		1.3×10^4^	
1974	16/06-14/10		01/06-31/10			1.4×10^5^		5.3×10^5^		6.9×10^4^		3.8×10^5^		1.1×10^6^		2.3×10^5^		1.9×10^5^		1.0×10^5^	
Total						5.9×10^5^		6.3×10^6^		5.9×10^5^		5.1×10^6^		2.7×10^6^		1.0×10^6^		1.5×10^6^		1.3×10^6^	

^a^, Dates are given for New Zealand, which are +1 day to French Polynesia dates; ^b^, Average for Auckland, Wellington and Christchurch in 1966-1968, for Auckland, Wellington, Christchurch and Hokitika in 1970-1974

**Table 3:** Time-Integrated ^131^I Activity Concentration in Cow’s Milk Locally Produced in Oceania, South America, Madagascar, and French Polynesia in 1964-1974

Year	Time-integrated ^131^I activity concentration in locally produced cow’s milk (Bq d L^-1^)											
	Fiji (Suva)	Samoa (Apia)	New Zealand^a^	New Caledonia (Noumé^a^)	Australia^a^	Ecuador (Quito)	Peru (Lima ^b^)	Colombia (Bogotá)	Chile (Santiago)	Bolivia (La Paz)	Madagascar (Antsiranana)^c^	French Polynesia (Tahiti)
1966	210	150	18-43	–^d^	56-410	93	220	–	150	–	480	520^ e^
1967	41	230	0.56-11	23	14-380	< LD	180	15	35	–	77	240
1968	150	130	5.4-29	< LD^ f^	29-170	20	150-370 ^g^	56	–	–	240	190
1970	130	240	7.6-46	130	32-210	< LD	30-280 ^h^	33	91	440	–	490
1971	92	250	1.9-16	96	13-200	< LD	110^ i^	28	190	89	240	650
1972	2	0.64	<0.074-1.5	< LD	1.9-6.3	< LD	< LD ^i^	10	70	14	< LD	40
1973	11	39	0.22-1.9	< LD	1.3-10	< LD	–	11	18	180	< LD	410
1974	96	150	4.6-25	< LD	13-91	48	580 ^i^	10	78	110	–	2400

^a^, Range of values measured in monitoring stations; ^b^, Lima unless otherwise indicated; ^c^, Zebu’s milk; ^d^, No measurements were reported; ^e^, Estimated based on reconstructed fallout; ^f^, Less than limit of detection; ^g^, Range of values measured in Lima, Arequipa and Tacna; ^h^, Range of values measured in Lima, Huancayo and Arequipa; ^i^, Arequipa.

**Table 4:** Comparison of Annual Radiation Doses from Atmospheric Nuclear Weapon Tests Conducted at Mururoa and Fangataufa Atolls in 1964-1974 to populations of Australia, New Zealand, South America, Africa and French Polynesia

Year	Whole-body dose (mGy) due to external irradiation			Thyroid dose (mGy) due to ^131^I intake with cow’s milk for 1-y old child							
	Australia ^a,b^	French Polynesia (Tahiti) ^c^	Australia^ b^	New Zealand ^b^	Ecuador (Quito)	Peru ^d^	Colombia (Bogotá)	Chile (Santiago)	Bolivia (La Paz)	Madagascar (Antsiranana)^f^	French Polynesia (Tahiti)
1966	4.4×10^-3^	0.032	0.43	0.17	0.03	0.03	–	0.05	–	0.2	0.69
1967	1.0×10^-3^	0.02	0.1	0.05	<0.01	0.1	0.02	0.04	–	0.09	0.32
1968	2.8×10^-3^	9.5×10-3	0.16	0.08	<0.05	0.35	<0.05	–	–	0.3	0.25
1970	3.3×10^-3^	0.015	0.15	0.08	<0.01	0.03^ e^	<0.01	0.05	0.15	–	0.65
1971	7.1×10^-3^	0.012	0.13	0.05	< 0.02	0.05 ^e^	< 0.02	0.1	0.05	0.01	0.87
1972	< 1.0×10^-3^	3.6×10-3	3.0×10^-3^	0.05	<0.01	<0.01	<0.01	0.04	<0.01	<0.01	0.053
1973	3.0×10^-4^	0.01	8.0×10^-3^	0.05	<0.01	–	<0.01	<0.01	0.08	<0.01	0.55
1974	5.3×10^-3^	0.34	0.09	0.05	0.02	0.24 ^e^	<0.01	0.03	0.05	–	3.2

^a^, Estimates without account to shielding properties of buildings and environment; ^b^, Country-wide population-weighted average dose; ^c^, Estimates accounting for the shielding properties of buildings and environment; ^d^, Lima unless otherwise indicated; ^e^, Arequipa; ^f^, Due to consumption of zebu’s milk.

**Table 5:** Comparison of Thyroid Doses for 1-y Old Child and Adult Person after Test Centaure on 17 July 1974

Parameter	Fiji (Suva)	Samoa (Apia)	Tonga (Tongatapu)	Cook Islands (Rarotonga)	French Polynesia (Tahiti)
Time-integrated total β-concentration air (Bq s m^-3^)	9.2×10^4^	2.3×10^6^	7.0×10^4^	8.1×10^5^	5.5×10^7^
Time of arrival	H+4d	H+4d	H+9d	H+3d	H+56h
Precipitation	Yes	No	Yes	Yes	Yes
Deposition density (Bq m^-2^):					
Total	5.7×103	4.1×10^4^	4.3×10^3^	5.0×10^4^	3.4×10^6^
^131^I	250	1.8×10^3^	350	1.7×10^3^	9.5×10^4^
Time-integrated ^131^I activity concentration in fresh cow’s milk (Bq d L^-1^)	38	130	–	–	2,300
Thyroid dose to 1-y child (mGy) due to:					
Inhalation of ^131^I and short-lived ^132^I,^ 133^I, ^135^I, ^132^Te	5.4×10^-4^	0.013	5.3×10^-4^	4.3×10^-3^	0.28
Ingestion of ^131^I with fresh cow’s milk	0.051	0.17	–	–	3.1
External irradiation	9.6×10^-4^	6.3×10^-3^	1.3×10^-3^	6.7×10^-3^	0.39
Total	0.052	0.19	1.8×10^-3^	0.011	3.8
Thyroid dose to adult (mGy) due to:					
Inhalation of ^131^I and short-lived ^132^I, ^133^I, ^135^I, ^132^Te	2.1×10^-4^	5.2×10^-3^	2.2×10^-4^	1.6×10^-3^	0.1
Ingestion of ^131^I with fresh cow’s milk	5.1×10^-3^	0.018	–	–	0.31
External irradiation	8.0×10^-4^	5.4×10^-3^	1.1×10^-3^	5.7×10^-3^	0.33
Total	6.1×10^-3^	0.028	1.3×10^-3^	7.3×10^-3^	0.97

## Discussion

To the authors’ knowledge, this study is the first to evaluate the potential radiological impact of atmospheric nuclear weapons tests conducted in 1966-1974 at Mururoa and Fangataufa atolls on populations in Oceania, South America and Africa. This evaluation is based on the analysis of the results of measurements of total β-concentrations in filtered air and of the ^131^I activity concentrations in locally produced cow’s milk, as well as radiation doses due to external irradiation and thyroid doses due to ^131^I intake with milk by local populations in different countries in the southern hemisphere. However, there are two factors that complicate the analysis: 

1. For the three monitoring stations in South America that are located in the 10-40^o^ latitude South band (La Paz, Lima, and Santiago) where the ‘primary’ cloud was generally found, the average TOAs were shorter than those for all monitoring stations in Oceania, except for Tahiti, although their distances to the test sites were greater, except for Australia. This is presumably due to the fact that the air contamination at those three monitoring stations was generally due to the ‘primary’ cloud, blowing from East to West, whereas the air contamination in Oceania was usually due to ‘secondary’ clouds blowing from West to East. The time-integrated β-concentrations in air, summed for all years of atmospheric testing, are greater in La Paz, Lima, and Santiago than in any monitoring station in Oceania, except for Tahiti and Samoa (Table 2). For specific years or specific time periods, the time-integrated air concentrations in Tahiti were even lower than those in La Paz, Lima, or Santiago. For example, the total β-concentration in air was almost twice higher in 1970 in Peru (Lima) and in 1972 in Chile (Santiago) than that in Tahiti during corresponding years. 

2. For a given test and a given, or similar, TOA, the total β-concentrations in air could vary substantially from a location to another. Taking as an example the test Pallas, detonated on 18 August 1973, ‘secondary’ clouds led to the contamination of part of Oceania. Practically the same total β-concentrations in air were measured in Samoa and Tahiti, with maximums of about 4 Bq m^-3^ and 1 Bq m^-3^, respectively. However, these concentrations were much lower than those measured at Raivavae (110 Bq m^-3^) and Tubuai (170 Bq m^-3^) on 22 August 1973. It seems reasonable to assume that the air contamination at the 4 monitoring stations was due to 3 separate air masses that moved in the same general direction: (1) the ‘Tahiti’ air mass, with a peak of contamination around 21 August 1973 (0.85 Bq m^-3^), (2) the ‘Samoa’ air mass, with a peak of contamination on 23 August 1973 (4.3 Bq m^-3^), and (3) the ‘Raivavae-Tubuai’ air mass, resulting in a peak of contamination on 22 August 1973 (>100 Bq m^-3^). 

In comparison to Tahiti, higher time-integrated ^131^I activity concentrations in cow’s milk could be expected for some locations in South America because of the typical trajectories of the ‘primary’ cloud. However, they were not expected for Australia. The high ^131^I activity concentrations in milk observed for Madagascar can be explained by the fact that milk from zébu was monitored. Lower zebu’s milk productivity, 0.8‒1.4 L d^-1^, in comparison with that for cows (Guillermo, 1949) resulted in much higher ^131^I activity concentrations in zebu’s milk than in cow’s milk.

Our study provides a comprehensive analysis of the available information on the radioactive contamination of the environment collected in Oceania, South America and Africa during the testing period in French Polynesia. There are, however, some limitations in this study. First, the available results of radiation monitoring in some countries were less detailed than those available for French Polynesia. Second, measurements of radioactive contamination were not reported for some locations, especially during the first years of the testing period (see Tables 1‒3). These limitations resulted in an incomplete evaluation of the radiological impact of the French nuclear tests to the populations of some countries; however, to the best of our knowledge, these limitations do not change the overall conclusions of the study. 

In conclusion, published results of radiation monitoring in Oceania, South America and Africa during the 1966-1974 time period of atmospheric nuclear weapons tests at Mururoa and Fangataufa atolls provided independent validation of radiation measurements done during the same time period in French Polynesia. The total β-concentrations in filtered air measured in French Polynesia were, in general, higher than those monitored in other locations in southern hemisphere by factors varying from 10 to 800. ^131^I activity concentrations in locally produced milk and radiation doses to the thyroid glands of local population were also higher in French Polynesia for most years at most monitoring locations in the southern hemisphere. However, for some years during the testing period, the total β-concentrations in filtered air and ^131^I activity concentrations in locally produced milk in South America were found to be similar or slightly higher than those measured in Tahiti. Thyroid radiation dose received outside French Polynesia by children from fallout after French nuclear tests were lower than 1 mGy. The health effect of French atmospheric nuclear weapons tests at Mururoa and Fangataufa on populations outside French Polynesia, which received doses lower than the ones from the natural source of radiation, is, if existing, very low, i.e. about 1 case of thyroid cancer in 10 years in 100,000 persons.

## Author Contribution Statement

The authors confirm contribution to the paper as follows: study conception and design: V. Drozdovitch, A. Bouville; data collection: F. de Vathaire, A. Bouville; analysis and interpretation of results: V. Drozdovitch, F. de Vathaire, A. Bouville; draft manuscript preparation: V. Drozdovitch, F. de Vathaire, A. Bouville. All authors reviewed the results and approved the final version of the manuscript.
